# Evaluation of SAS1B as a target for antibody-drug conjugate therapy in the treatment of pancreatic cancer

**DOI:** 10.18632/oncotarget.23944

**Published:** 2018-01-04

**Authors:** Kiley A. Knapp, Eusebio S. Pires, Sara J. Adair, Arabinda Mandal, Anne M. Mills, Walter C. Olson, Craig L. Slingluff, J. Thomas Parsons, Todd W. Bauer, Timothy N. Bullock, John C. Herr

**Affiliations:** ^1^ Department of Pathology, The School of Medicine, University of Virginia, Charlottesville, Virginia, USA; ^2^ Department of Cell Biology, The School of Medicine, University of Virginia, Charlottesville, Virginia, USA; ^3^ Department of Obstetrics and Gynecology, The School of Medicine, University of Virginia, Charlottesville, Virginia, USA; ^4^ Department of Surgery, The School of Medicine, University of Virginia, Charlottesville, Virginia, USA; ^5^ Department of Microbiology, The School of Medicine, University of Virginia, Charlottesville, Virginia, USA

**Keywords:** ASTL/SAS1B/ovastacin, antibody-drug conjugate, pancreatic cancer biomarker, surface cancer-oocyte antigen, targeted immunotherapy

## Abstract

Successful therapeutic options remain elusive for pancreatic cancer. The exquisite sensitivity and specificity of humoral and cellular immunity may provide therapeutic approaches if antigens specific for pancreatic cancer cells can be identified. Here we characterize SAS1B (ovastacin, *ASTL*, astacin-like), a cancer-oocyte antigen, as an attractive immunotoxin target expressed at the surface of human pancreatic cancer cells, with limited expression among normal tissues. Immunohistochemistry shows that most pancreatic cancers are SAS1B^pos^ (68%), while normal pancreatic ductal epithelium is SAS1B^neg^. Pancreatic cancer cell lines developed from patient-derived xenograft models display SAS1B cell surface localization, in addition to cytoplasmic expression, suggesting utility for SAS1B in multiple immunotherapeutic approaches. When pancreatic cancer cells were treated with an anti-SAS1B antibody-drug conjugate, significant cell death was observed at 0.01-0.1 μg/mL, while SAS1B^neg^ human keratinocytes were resistant. Cytotoxicity was correlated with SAS1B cell surface expression; substantial killing was observed for tumors with low steady state SAS1B expression, suggesting a substantial proportion of SAS1B^pos^ tumors can be targeted in this manner. These results demonstrate SAS1B is a surface target in pancreatic cancer cells capable of binding monoclonal antibodies, internalization, and delivering cytotoxic drug payloads, supporting further development of SAS1B as a novel target for pancreatic cancer.

## INTRODUCTION

Pancreatic cancer continues to pose a serious clinical challenge, being the fourth leading cause of cancer-related deaths in the United States. Total deaths due to pancreatic cancer are predicted to increase dramatically, with the expectation that it will become the second leading cause of cancer-related deaths by 2030 [1]. Pancreatic ductal adenocarcinoma (PDAC) originates in the exocrine pancreas and accounts for 95% of all pancreatic cancers [2]. The overall five-year survival rate has remained resistant to improvement, from 2% in 1975 to only 8% currently; for most pancreatic cancer patients, life expectancy is measured in months [3, 4]. Conventional treatment approaches, such as surgery, radiation, chemotherapy, or a combination of these, have had little impact on this aggressive tumor due to: 1) late stage diagnosis, which precludes surgery as a viable option, 2) lack of effective early detection biomarkers, 3) early and frequent metastases, and 4) eventual therapeutic resistance [5–7]. The need for effective, novel treatments for PDAC is clear.

Recent advances in the treatment of metastatic disease using combination chemotherapeutics have only increased overall survival in terms of months [8, 9]. The dense desmoplastic tumor stroma, characteristic of PDAC, contributes to inadequate therapeutic penetration and promotes resistance to chemo- and radiotherapy [6, 10]. However, combination of these more aggressive chemotherapies with therapies that engage novel targets may represent a promising therapeutic strategy for the treatment of PDAC [6]. Molecular therapies targeting specific, novel cancer targets, including passive approaches such antibody-drug conjugates (ADC) and chimeric antigen receptor T cells (CAR-T) or active approaches like cancer vaccines, have been given widespread attention in hopes of developing more specific and less toxic therapeutics [5, 11]. ADCs represent a subset of immunotherapies whose functional activity is not dependent upon targeting oncogenic pathways that drive tumorigenesis, but via the recognition of proteins that are uniquely expressed by tumors, leading to direct or indirect immune-mediated destruction. Notably, two FDA approved ADCs, brentuximab vedotin (Adcetris) targeting CD30 and trastuxamab emtansine (Kadcyla) targeting Her2, have revolutionized treatment for Hodgkin’s lymphoma and breast cancer, respectively [12] and have helped promote greater effort to identify and validate novel targets for ADCs.

SAS1B (sperm acrosomal SLLP1 binding protein; aka ovastacin, *ASTL*, GenBank ID NM_001002036.3) is an oocyte membrane-associated zinc metalloprotease binding partner for a sperm ligand which contributes to fertilization [13, 14] and also plays a role in the block to polyspermy [15]. SAS1B, a 46 kDa protein, is comprised of a signal peptide at the N-terminus, pro-peptide, proteinase domain containing a Hex-box catalytic site, and a unique C-terminal domain. Among normal tissues, SAS1B expression is limited to the ovary, specifically to oocytes at or beyond the secondary follicle stage, and is absent in the quiescent ovarian reserve [14]. SAS1B was not detected in a variety of additional normal human tissues from a tissue microarray (TMA) by immunohistochemistry (IHC) [16] and no *ASTL* expressed sequence tags have been deposited in NCBI Unigene database from normal tissues [17].

In addition to localization within the ovary, SAS1B has also been shown to be expressed in a majority uterine tumors (66–85%) [16]. In a uterine tumor cell line SNU539, SAS1B was shown to be localized to the cell membrane, internalized via the endocytic pathway, and sensitive to growth arrest and cell death in the presence of an indirect antibody-saporin (drug) conjugate using a rabbit polyclonal antibody targeting SAS1B [16]. SAS1B represents one of the first defined antigens in a new class of cancer-oocyte antigens [16]. Cancer germline antigens (CGA), exemplified by the well-studied, numerous cancer testis antigens (CTA), are normally expressed discretely in germ cells and trophoblasts but are re-expressed in various human cancers [18, 19]. It is theorized that CGAs are aberrantly expressed in tumors when the silenced gametogenic program in somatic cells is activated, and that this program acquisition, in part, contributes to tumorigenesis [20, 21]. These studies position SAS1B as a viable target of an immunotoxin in cancer, with the attending advantages of limited on target/off-tumor effects on normal tissues, and support the study of ADCs for the treatment of SAS1B-positive (SAS1B^pos^) tumors.

The following study provides evidence that SAS1B is expressed in a majority of pancreatic cancers, is localized to the cell surface, and that pancreatic cancer cells are killed when treated with an anti-SAS1B ADC, validating SAS1B as a target for further pre-clinical development.

## RESULTS

### SAS1B is expressed in a majority of pancreatic cancers and is not detected in normal pancreas ductal epithelium by IHC

**Figure 1:**
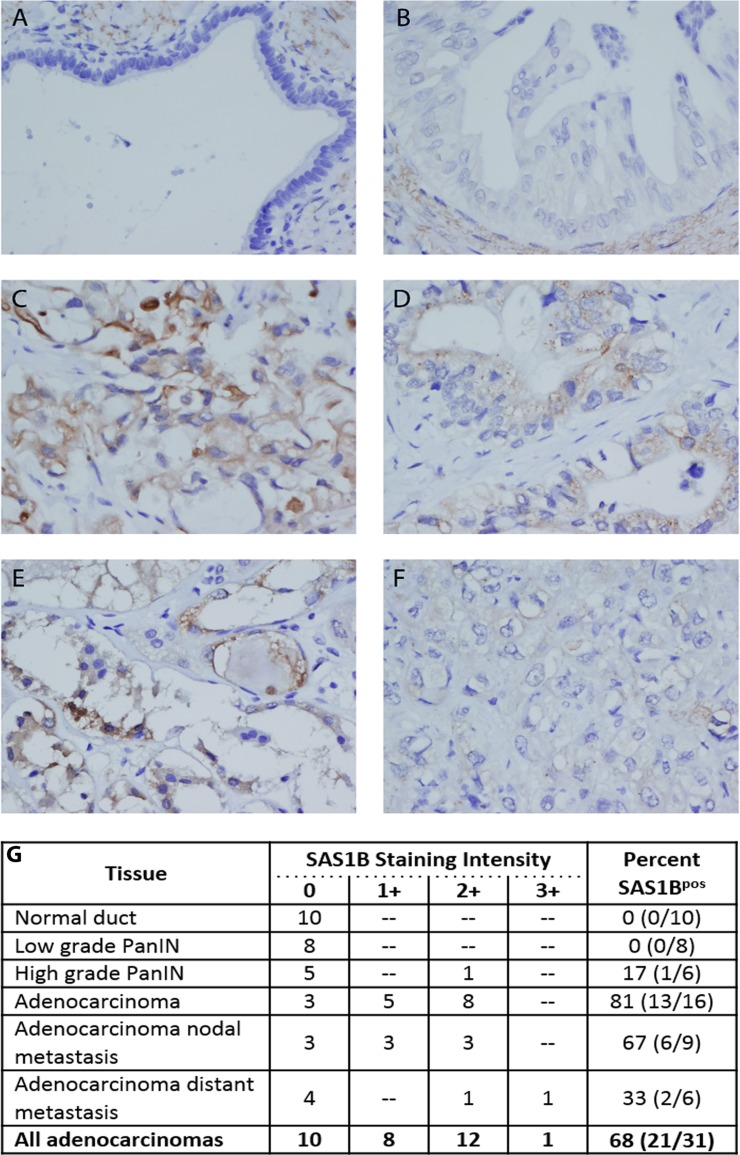
SAS1B was expressed in a majority of pancreatic cancers and was not detected in normal pancreas ductal epithelium by IHC TMAs were stained for the expression of SAS1B with 6B1 mAb. SAS1B was not detected in normal pancreatic ductal epithelium (**A**) and most pancreatic intraepithelial lesions (**B**). Some stromal cells adjacent to these ducts showed cytoplasmic reactivity, as pictured in A/B. Many ductal carcinomas showed cytoplasmic SAS1B staining (**C**–**E**). This ranged from strong, diffuse staining that also included some ill-defined membranous positivity (C) to focal, exclusively cytoplasmic staining (D-E). A minority of ductal carcinomas were negative or showed only trace non-specific staining (**F**). Images are 400× magnification. SAS1B staining was scored on a 0 (negative) to 3+ positivity scale for each tissue type and result are summarized in the table (**G**). Percent of samples that were SAS1B positive, for each tissue type, is quantified in the last column (total number of SAS1B positive samples/ total number of samples) (G).

Given the expression of *ASTL* (gene)/SAS1B (protein) in uterine cancer [16], we hypothesized that *ASTL*/SAS1B may be expressed in PDAC. Immunohistochemistry using an anti-SAS1B monoclonal antibody (mAb) (6B1; shown to largely recognize cytoplasmic SAS1B) was performed on a TMA containing primary and metastatic pancreatic cancer samples, pancreatic intraepithelial neoplasia (PanIN; precursor lesions), and normal duct from both benign and malignant pancreas. TMA staining results were read in a blinded manner and scored by a pathologist on a 0 (negative) to 3+ positivity scale. SAS1B staining was not detected in untransformed ductal epithelium present in either benign or malignant pancreas (*n* = 10) (Figure 1A–1B). Low-grade PanINs were also SAS1B negative (*n* = 8). SAS1B staining was observed in one out of six high grade PanINs. In some cases, stromal cells adjacent to ducts in normal and low grade tumors showed weak cytoplasmic reactivity (Figure 1A–1B).

In contrast to the limited staining in low grade tumors, the majority of PDACs were SAS1B^pos^ (68%, *n* = 21/31), (Figure 1C–1E). Both primary (*n* = 13/16) and metastatic (*n* = 8/15) tumors were SAS1B^pos^. Most cancers exhibited 1+ or 2+ SAS1B staining intensity. When 6B1 mAb was pre-incubated with recombinant SAS1B (rSAS1B) protein and then added to histology sections, no staining was detected (Supplementary Figure 1). Staining of PDACs was cytoplasmic in all cases while membranous localization was also observed in a few cases. Positive staining could be characterized across a range from strong, diffuse staining that included some ill-defined membranous staining (Figure 1C) to focal, exclusively cytoplasmic staining (Figure 1D–1E). Within individual tumors, SAS1B positivity ranged from about 10% to greater than 90% of cancerous cells staining. Approximately 30% of PDACs had no detectable SAS1B or showed only trace staining (Figure 1F). Importantly, expression of SAS1B was found both in primary tumors and in metastatic tumors from the lymph node and distal peripheral sites (Figure 1G). One of six high-grade PanIN samples were SAS1B^pos^, suggesting that SAS1B expression may first appear in advanced precursor lesions during carcinogenesis. These data demonstrate SAS1B is expressed in a majority of pancreatic cancers evaluated and is not detected in normal human pancreatic ductal epithelium, providing rationale for further investigation of SAS1B as a therapeutic target for the treatment of PDAC.

### *ASTL*/SAS1B is expressed in pancreatic cancer patient derived xenografts

**Figure 2:**
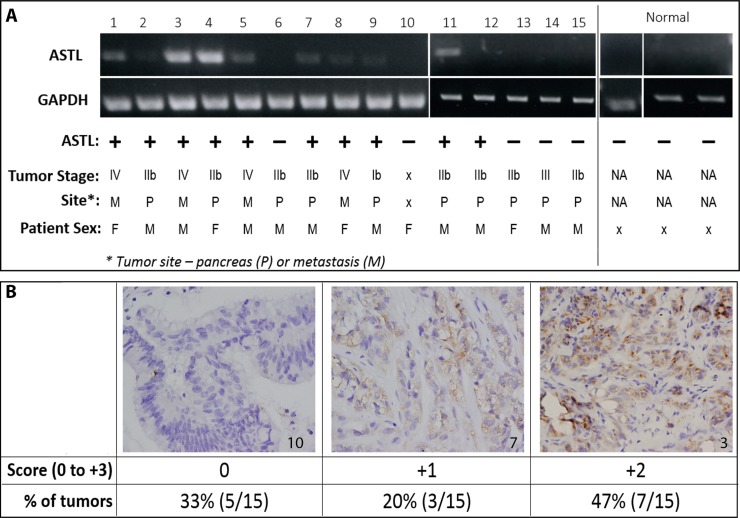
ASTL/SAS1B expression in pancreatic cancer patient derived xenografts (**A**) RT-PCR analyses of 15 PDAC (1-15) PDX tumors and 3 normal human pancreas (normal) samples using a c-terminus ASTL specific primer set showed a 309 bp amplicon in 10/15 PDAC samples. Tumors from both males and females, early and late stage disease, as well as primary and metastatic tumors were ASTL^pos^ (Table). GAPDH was used as a housekeeping control for PCR. (**B**) Immunohistochemical localization of SAS1B, on representative examples from the same set of 15 PDAC tumors used in (A), labeled with anti-SAS1B mAb, 6B1. Tumor number indicated in bottom right corner of image. Images are 400× magnification. Tumors were scored on a 0 (negative) to 3+ positivity scale; total number of tumors in each group quantified in the table.

With the intent of identifying potential *in vivo* models that could be used to develop and to assess SAS1B-specific targets for therapeutic and diagnostic approaches, we evaluated SAS1B expression in patient derived xenografts (PDX). Tumors were obtained from PDAC PDX mouse models that have been previously shown to have high genotypic and phenotypic concordance with the source patient tumor. These PDAC PDX orthotopic models, where fresh patient tumors are affixed directly into the mouse pancreas, have been shown to recapitulate the clinical, pathological, genetic, and molecular aspects of human disease and are thus regarded as superior, clinically-relevant models [22]. RNA was isolated from 15 tumors and 3 normal human pancreas samples and was reverse-transcribed to cDNA. PCR analysis using a primer for the c-terminus of *ASTL* set showed no detectable *ASTL* transcript in normal pancreas; however, 67% (10/15) of PDAC tumors were positive for *ASTL* transcript (Figure 2A). Amplicons were cloned and sequenced, revealing 99% identity to *ASTL* reference sequence with an occasional single nucleotide polymorphism.

Immunohistochemical staining for SAS1B expression was performed on these PDX tumor samples; 67% (10/15) were positive for SAS1B protein (Figure 2B), with each tumor’s protein expression concordant with *ASTL* transcript data shown in Figure 2A. Tumors were scored by a pathologist, in a blinded manner, on a 0 (negative) to 3+ positivity staining-intensity scale. SAS1B was largely localized intracellularly with 1+ and 2+ staining intensities. Correlation of *ASTL*/SAS1B expression with patient data showed that *ASTL*/SAS1B expression occurs in tumors from both males and females, tumors of early and late stage (II to IV), as well as in primary and metastatic tumors (Figure 2A). These data show agreement between *ASTL* transcript and SAS1B message within all pancreatic tumors examined, suggesting SAS1B translation occurs with a high degree of concordance with *ASTL* transcription. The robustness of PCR bands (e.g. tumors numbered 3 & 4) correlated with stronger IHC staining intensity (2+). The 67% incidence among this cohort of 15 PDX cancer samples matches the 68% incidence identified in the human cancer samples (Figure 1G). These data show that SAS1B expression is maintained when primary human tumors are grafted into immunodeficient mice, highlighting the potential utility of this model for *in vivo* development.

### SAS1B localizes to the cytoplasm and to the cell surface of pancreatic cancer cell lines

**Figure 3:**
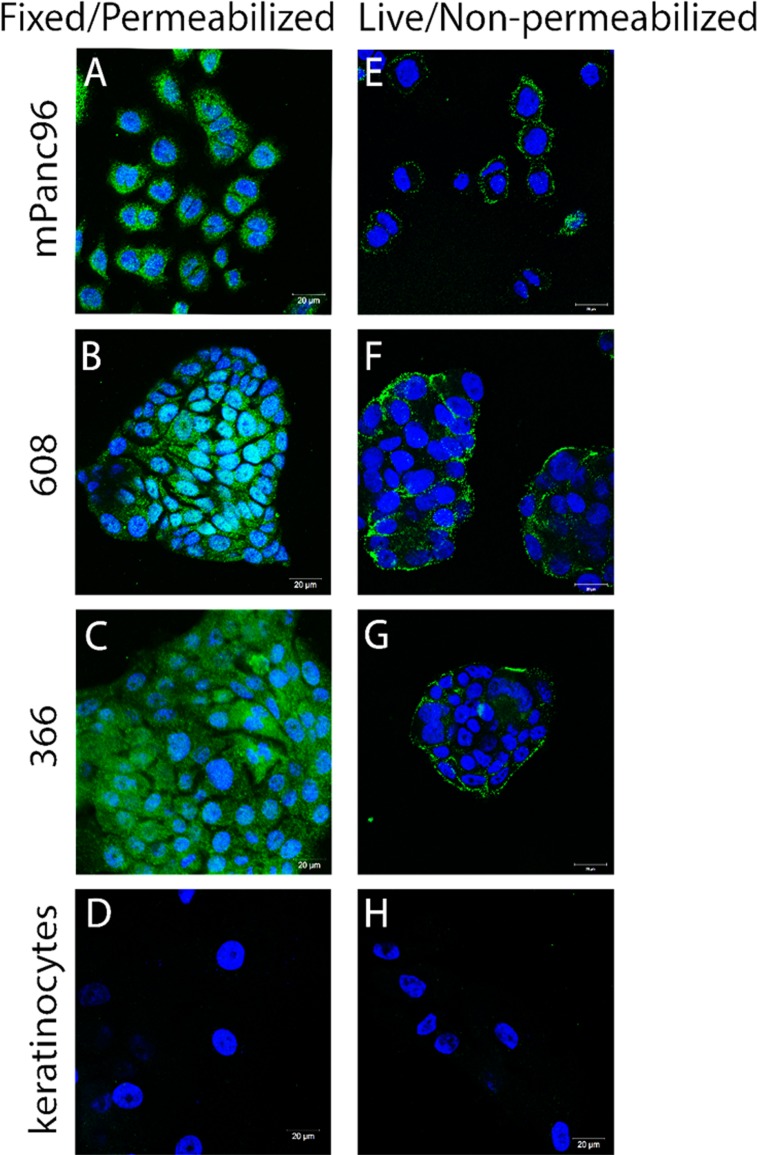
SAS1B localized to the cytoplasm and to the cell surface in pancreatic cancer cell lines (**A**–**D**) Fixed and permeabilized indirect immunofluorescence (IIF) using anti-SAS1B mAb, SB2 showed SAS1B localized to the cytoplasm of three pancreatic cancer cell lines (mPanc96, 366, 608) compared to normal keratinocytes. (**E**–**H**) IIF on live, non-permeabilized cells using anti-SAS1B mAb, SB2, demonstrated staining of the plasma membrane of mPanc96, 366 and 608 cells but not normal keratinocytes. Data are representative of three independent experiments.

The IHC analyses raise the possibility that SAS1B may be expressed at the cell surface of some pancreatic cancer cells, but routine IHC is not sufficient to confirm cell surface expression. SAS1B has a putative transmembrane sequence; thus, we hypothesized that cell-surface expression of SAS1B may be sufficient to support therapeutic approaches with ADCs and/or CAR-T cells. To obtain preclinical data to address this question, three pancreatic cancer cell lines (mPanc96, 366, 608) were further evaluated *in vitro* with confocal analysis. Cell lines 366 and 608, which are patient derived and match tumors numbered 3 and 5, respectively (Figure 2), were chosen because they are known to highly recapitulate the patient tumor [22]. Unlike 366 and 608, mPanc96 was chosen because it is a cell line that has been substantially characterized in a variety of pancreatic cancer studies. Confocal analysis of indirect immunofluorescence (IIF) of fixed and permeabilized pancreatic cancer cells, using anti-SAS1B mAb SB2, showed that SAS1B is abundant in the cytoplasm (Figure 3A–3C). No signal was detected with non-specific mouse IgG antibody (data not shown). These IIF data using SB2 mAb are in agreement with the IHC data indicating a prominent cytoplasmic localization of SAS1B (Figures 1 and 2). To determine if SAS1B is also expressed at the cell surface, IIF on live, non-permeabilized cells was performed. IIF on live pancreatic cancer cells, using the SB2 anti-SAS1B mAb, shows that SAS1B is present at the cell membrane in a majority of cells in a punctate surface staining pattern (Figure 3E–3G). No detectable SAS1B was observed in non-neoplastic keratinocytes (Figure 3D, 3H).

The majority of pancreatic cancer cells in all three cell lines express cytoplasmic and cell surface SAS1B. Cell lines 608 and 366 grow in clumps/clusters, and in these samples, the most robust live cell staining was observed at the periphery of the cell cluster. However, when Z-stack analysis was used in confocal microscopy, punctate SAS1B signal was observed across the surface of the cells in the interior of the cell cluster (data not shown). Taken together, these data suggest that while SAS1B expression appears more robust in the cytoplasm, a majority of cells also express a pool of cell surface SAS1B. As SAS1B expression in mPanc96, 366, and 608 cell lines can be found at the cell surface, we proceeded to test the ability of SAS1B to serve as a target for ADC-mediated killing.

### SAS1B surface expression in pancreatic cancer cell lines correlates with anti-SAS1B ADC cell killing *in vitro*

**Figure 4:**
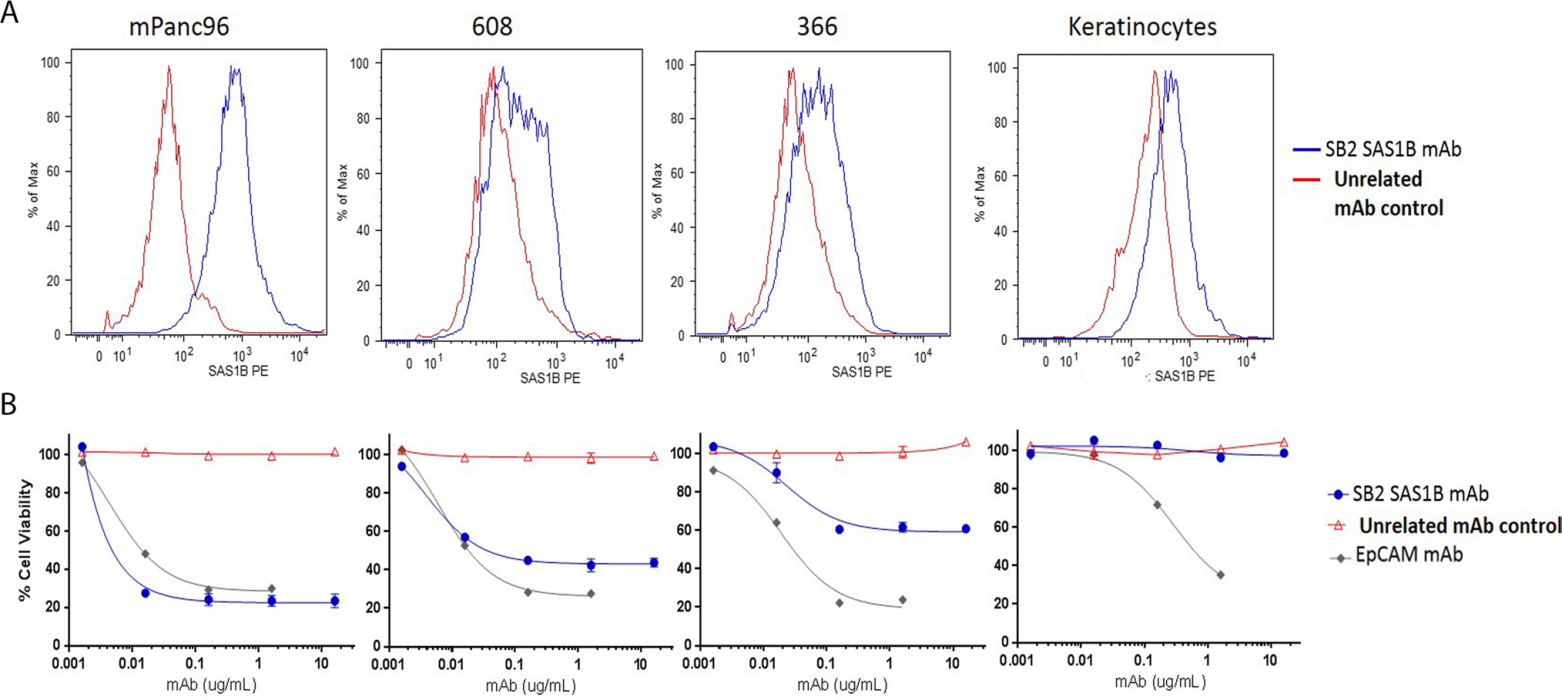
SAS1B surface expression in pancreatic cancer cell lines correlated to anti-SAS1B ADC cell killing *in vitro* (**A**) Median fluorescent intensity of cell surface SAS1B detected by live cell flow cytometry with SB2 monoclonal antibody (blue line) or unrelated control antibody (red line) on PDAC cell lines mPanc96 (left), 608 (left middle), and 366 (right middle) and keratinocytes (right). (**B**) Cytotoxicity by anti-SAS1B ADC (mAb SB2) titration shown below each flow-cytometric plot for corresponding cell line. SB2- mAb-Duocarmycin immune complexes were generated (ADC) then incubated with cells for 72 hours. Relative cell viability was measured using CellTiter-Glo. Data represent averages of three independent replicates, with 3 technical replicates in each data point.

Because SAS1B localizes to the cell membrane in pancreatic cancer cells and previous data have shown that SAS1B is endocytosed [16], we hypothesized that these could be killed using an ADC targeting SAS1B, with varying degrees of cytotoxicity correlated to relative antigen level at the cell surface. To first quantify the SAS1B surface expression observed in live IIF (Figure 3E–3H), relative amounts of cell surface SAS1B were detected by flow cytometry with SB2 performed on live pancreatic cancer cell lines (mPanc96, 366, 608) and normal keratinocytes (Figure 4A). Examination of relative proportions of SAS1B^pos^ cells revealed mPanc96 had the highest proportion of cells expressing SAS1B and the highest per cell SAS1B expression. 608 had intermediate expression, and 366 showed the weakest expression of SAS1B. Surprisingly, given the previous IIF data, keratinocytes also had a population of SAS1B^pos^ cells. No immunoreactivity was observed when SB2 mAb was pre-incubated with rSAS1B protein for one hour before adding to cells (Supplementary Figure 2).

To evaluate the SAS1B antibody SB2 as a candidate targeting immunotherapeutic drug, an *in vitro* cytotoxicity assay was performed, in which cells were incubated with SB2 complexed with a secondary antibody conjugated to the DNA-alkylating agent duocarmycin DM via a pH sensitive linker (Figure 4B). Cell death from a SAS1B-ADC is induced by disruption of DNA architecture from duocarmycin treatment. Total cellular ATP was measured using CellTiter Glo in a luminometer to determine percent viability of cells. No cytotoxicity was observed when cells were treated with secondary-drug conjugate alone as compared to cells without treatment (media only; Supplementary Figure 3B). Significant cell death was observed in mPanc96 and 608 cells treated with sub-nanomolar concentrations (0.01 nM, equivalent to 0.0016 μg/mL) and in 366 treated with nanomolar concentrations (1 nM, equivalent to 0.016 μg/mL) of anti-SAS1B mAb SB2 complexed with drug conjugate, as compared to the negative control ADC. At 0.016 μg/mL SB2, a statistically significant difference in cytotoxicity was observed when comparing keratinocytes to each of the three PDAC cell lines (mPanc96, 608, 366) independently (*t*-test *p*-value < 0.0001; ANOVA *p*-value < 0.0001). The LD50 values for mPanc96 were 0.0055 ± 0.002, 608 were 0.0088 ± 0.006, and 366 were 0.011 ± 0.005 μg/mL SB2 mAb. Although mPanc96 trended toward being more effectively killed, there were no significant differences in the LD50 between the three cancer cell lines (*n* = 3 assays; non-linear regression analysis). Similar results, in terms of general pattern of killing and concentration at which cell death is observed, were found with additional SAS1B-specific mAbs (data not shown). In the absence of drug-conjugate, anti-SAS1B mAb SB2 alone was not cytotoxic to cells (Supplementary Figure 3A), warranting the need for a cytotoxic agent since prior heat inactivation of serum in complete media prevented complement mediated cell death. The drug-conjugate, in the absence of SB2, also showed no cytotoxic effects (data not shown). SB2 mAb killing specificity was confirmed by the lack of cytotoxicity with antibody that had been pre-incubated with SAS1B blocking peptide while no effect on cytotoxicity was observed when using an SAS1B peptide which is not recognized by SB2 (Supplementary Figure 4). Keratinocytes did not stain for the target by IIF, and despite observation of some SAS1B signal in flow cytometry, no *in vitro* cytotoxicity using anti-SAS1B-ADC was observed in these cells. Similar cytotoxic effects were observed among all three pancreatic cancer cell lines using a positive control ADC targeting epithelial cell adhesion molecule (EpCAM) (Figure 4B). This demonstrates that the differential cytotoxic responses observed with anti-SAS1B ADC are as a result of properties of the target rather than inherent differences in how these cells process an ADC or differential resistance to apoptosis.

Notably, mPanc96 and 366 cells had the highest and lowest SAS1B surface expression by flow cytometry and most and least ADC mediated cell killing as a proportion of the treated population, respectively. Thus, the difference in absolute killing apparent in Figure 4 reflects the amount of protein expressed at the cell surface. These data suggest potential utility of stratifying patients based on the level of SAS1B surface expression with regard to treatment with anti-SAS1B therapies. These results demonstrate SAS1B is a surface target in pancreatic cancer cells capable of binding monoclonal antibodies, internalization, and delivering cytotoxic drug payloads which effectively kill cancer cells.

## DISCUSSION

### SAS1B is a newly identified cancer-oocyte antigen in pancreatic cancer

Here we report that SAS1B is expressed in a majority of both patient PDACs and PDX PDAC samples. We find that SAS1B localizes to both the cytoplasm and the cell surface in PDAC cell lines. Further, we validated SAS1B as a therapeutic target by determining that co-incubation of pancreatic cancer cell lines with anti-SAS1B ADC results in cytotoxicity *in vitro*, indicating that SAS1B has characteristics of a bone fide targetable antigen for pancreatic cancer.

Among a cohort of 31 pancreas cancer samples, 68% were SAS1B^pos^ by IHC; SAS1B expression was observed in both primary and metastatic tumors (Figure 1). Increased staining intensity was observed with advanced disease indicative of increased SAS1B molecule density, however, the sample size was small. Further studies with increased sample size would help statistically correlate SAS1B expression intensity and frequency with disease characteristics. These data do suggest a relatively high penetrance of expression but a heterogeneity in both the overall frequency of expression and proportion of tumor cells that express SAS1B, indicating that careful pathological assessment will need to be performed prior to therapy selection. Moreover, while Pires *et al*. showed SAS1B expression in cancers from the female reproductive tract (precisely uterine tumors) [16], this is first paper to report SAS1B^pos^ cancers from males, which is notable since, in untransformed tissue, SAS1B is localized to ovaries in females [16]. The expression of SAS1B in male tumors, but not in male normal tissue, may mean that immune tolerance to SAS1B is not well established in males, making it an even more attractive target. However, this conclusion is dependent upon the verification of the low level of expression observed in tumor stroma, and whether stromal expression is constrained to the tumor microenvironment and is absent from true normal pancreas.

SAS1B is expressed in the cytoplasm of pancreatic cancer cells by IHC (Figures 1 and 2), indirect immunofluorescence (Figure 3A–3C), and intracellular flow cytometry (Supplementary Figure 5). A population of SAS1B traffics to the plasma membrane and is accessible on the cell surface of pancreatic cancer cells (Figures 3 and 4). The punctate staining pattern observed at the cell surface suggests that SAS1B may be associated with lipid rafts or sites of exocytosis. The cycling rates and half-life of SAS1B at the plasma membrane are currently unknown but the data presented here suggest that there is a greater pool of SAS1B in the cytoplasm and that a fraction of the total translated SAS1B traffics to the cell surface. While IIF and flow cytometry on fixed and permeabilized cells suggests most cells express cytoplasmic SAS1B, IIF data on live, non-permeabilized cells indicates a greater pool of cells express cell surface SAS1B than shown by flow cytometry. The flow cytometry data may slightly underestimate SAS1B expression as the digestion procedure may cleave some SAS1B from the cell surface. However, a majority of cells express SAS1B at low levels in flow cytometry, despite it being a snapshot assessment of expression. The high degree of cytotoxicity observed with a SAS1B-ADC in these lines is consistent with greater surface expression of SAS1B than indicated by the flow cytometry assays. Trafficking of SAS1B to the membrane and how SAS1B expression relates to tumor biology and disease prognosis is unknown and requires additional studies.

### Potential diagnostic and imaging applications of SAS1B

SAS1B has been shown to be exocytosed from oocyte cortical granules after fertilization to aid in the block to polyspermy by cleaving zona pellucida protein 2 (ZP2) surrounding two-cell embryos [14, 15] but it is unknown whether SAS1B^pos^ cancer cells secrete SAS1B. Given these data, additional studies are warranted to determine whether shed SAS1B may be able to serve as a potential diagnostic marker in circulation for early detection of pancreatic cancer. Early detection of PDAC precursor lesions and of early stage disease could increase therapeutic opportunities and outcomes for patients. Further, this is the first study to report SAS1B expression in a precursor cancer lesion (Figure 1G); whether or not additional PanINs harbor SAS1B expression and if SAS1B contributes to carcinogenesis has yet to be determined. PanINs are understood to be noninvasive ductal precursor lesions to PDAC [23]. Within this study, all low grade PanINs were SAS1B^neg^ but one of six high grade PanINs was SAS1B^pos^. High-grade PanINs are also referred to as “carcinoma *in situ*” and many of the genes altered in invasive pancreatic cancer are also altered in PanINs [23, 24]. The finding of SAS1B expression in one high grade PanIN is provocative but insufficient to draw definitive conclusions about expression in PanINs. Nonetheless, futures studies assessing SAS1B expression in precursor lesions are warranted.

Over 80% of primary adenocarcinomas were SAS1B^pos^. Thus, there is potential for early detection of PDAC through screening of individuals with family history of pancreatic cancer using SAS1B as a biomarker, if SAS1B is shed at sufficient levels from cancer cells. Further, as SAS1B is expressed at the cell surface of tumor cells and rarely in normal tissue, there is the potential to develop cancer imaging applications with fluorescently or radio-labeled nanoparticles [25, 26]. A SAS1B targeted imaging approach has the potential to also serve as a way to stratify patients who would be most likely to respond to SAS1B therapies. However, high shed rates could also complicate the use of SAS1B for ADC targeting or imaging, as shed SAS1B could serve as an antibody sink.

### Evaluation of the therapeutic potential of SAS1B as a target for pancreatic cancer

SAS1B was shown to be internalized via the endocytic pathway in uterine tumor cells after antibody binding using a rabbit polyclonal antibody [16]. When pancreatic cancer cells were treated with complexes of SAS1B primary antibodies bound to secondary antibodies linked with the toxin duocarmycin DM, cell death was observed (Figure 4). This study shows that an ADC targeting SAS1B is internalized resulting in cytotoxicity of pancreatic cancer cells, thus supporting SAS1B as a candidate for ADC therapy.

The extent of anti-SAS1B ADC cell death observed correlates with the relative amount of surface-associated SAS1B within a given population of cells. mPanc96 and 366 cells were the highest and lowest SAS1B expressers by flow cytometry, respectively, and also showed the greatest and least amount of cell death (Figure 4). The equivalent cytotoxicity found between all three pancreatic cancer lines using a control ADC targeting EpCAM (Figure 4B) demonstrates that the differences observed among the cell lines with the anti-SAS1B ADC are due to target density differences rather than inherent differences in how each cell line processes and responds to an ADC. The flow cytometry data coupled with the cytotoxicity data shown in Figure 4 suggests that a limited pool of cell surface-associated SAS1B may be sufficient to induce cell death when SAS1B^pos^ pancreatic cancer cells are targeted with an anti-SAS1B ADC. Our data suggest that pancreatic cancer cells that have the highest density of SAS1B on the cell surface combined with the highest percentage of total cells being SAS1B^pos^ would be most likely to benefit the greatest from SAS1B targeted ADC therapy. However, in pancreatic cancer cell populations where less SAS1B is expressed, some cytotoxicity is still observed. Further studies are warranted to define the rate and regulation of SAS1B cycling to the plasma membrane to determine whether this influences the threshold of SAS1B expression needed to induce cancer cell death. This information could help guide the manipulation of surface SAS1B, and determine whether surface or intracellular expression needs to be assessed for the stratification of patients who would most likely respond to a SAS1B targeted ADC therapy.

Non-neoplastic keratinocytes showed a population of SAS1B^pos^ cells by live cell flow cytometry but were not affected by the anti-SAS1B ADC (Figure 4) suggesting that SAS1B-ADC complexes were not internalized in these cells as they were in PDAC cells (Figure 4). The discrepancy between the IIF data showing keratinocytes were SAS1B^neg^ (Figure 3D, 3H) and the flow cytometry data showing these cells were SAS1B^pos^ (Figure 4A), suggests that flow cytometry may be more sensitive than the IIF assay for SAS1B detection in keratinocytes. Alternatively, keratinocytes may express a different SAS1B isoform which is not internalized via the endocytic pathway; additional studies are required to determine whether or not this is the case.

Some stromal cells, from both normal and malignant pancreas, appeared to express SAS1B at a low level, localized to the cytoplasm, by IHC (Figure 1; Supplementary Figure 1). However, *ASTL* was not detected by RT-PCR in normal pancreas (Figure 2A). This discordance may be because the stromal component of the normal pancreas sections analyzed in Figure 2A may not have been great enough to allow for detection by PCR or that stromal cells associated with transformed tissue may be induced to express SAS1B. The latter result may further support targeting SAS1B as elimination of tumor stroma has been shown to be critical for the prevention of tumor recurrence. However, the physiological significance of SAS1B staining in stroma remains unknown. Given that non-neoplastic keratinocytes were shown to express SAS1B at the cell surface by flow cytometry but showed no cell death when treated with an anti-SAS1B ADC (Figure 4), we hypothesize that non-cancerous SAS1B^pos^ cells will not be affected by an anti-SAS1B ADC. This population of SAS1B^pos^ stromal cells requires further investigation and future studies using PDX xenografts will be necessary to ascertain the impact of stromal expression on the optimization of SAS1B-specific immunotherapeutic options.

The heterogeneity and mosaicism of SAS1B expression observed in PDAC has implications for immunotherapy selection. ADCs are an attractive option for SAS1B^pos^ PDACs because, based on the data presented, an anti-SAS1B ADC is cytotoxic to cancerous cells but not to non-neoplastic cells which is hypothesized to be related to differences in internalization. A cancer vaccine using SAS1B as a target is an additional immunotherapeutic option that warrants exploration. However, given that heterogeneity of SAS1B expression was observed, there may be a subpopulation of SAS1B^neg^ PDAC cells which are not targeted by a vaccine. It is likely that, even with a potential anti-SAS1B ADC therapy for the treatment of PDAC, combination with one or more additional therapies would provide the maximum anti-cancer benefit to patients.

Additional studies are warranted to further evaluate SAS1B as an ADC therapeutic target for the treatment of PDAC. The PDAC PDX mouse models examined in this study, where patient tumors are affixed directly into the mouse pancreas [22], may represent a suitable system for studying effects of anti-SAS1B therapies *in vivo*, along with assessment of SAS1B expression in the current murine genetic models of pancreatic cancers. The data presented in this study strongly suggest that SAS1B directed immunotherapies have the potential to provide a novel axis of therapy for the pancreatic cancer population.

## MATERIALS AND METHODS

### Antibodies and reagents

Mouse anti-SAS1B mAb 6B1 was selected from a hybridoma technology campaign to be able to screen for SAS1B^pos^ samples by IHC. For 6B1 [Pires *et al*., In Submission], a truncated human SAS1B immunogen [14] was used to inject mice and mAbs were generated by the Antibody Engineering and Technology (AbET) Core (University of Virginia (UVA)). For SB2, truncated SAS1B, lacking only the signal peptide, was expressed as described earlier [13] and was used as the immunogen to generate mouse anti-human SAS1B mAbs. Mouse anti-SAS1B mAb SB2 was selected from a hybridoma campaign based on a screen for SAS1B^pos^ live cancer cells. The mAbs were generated by the AbET Core (UVA). Unrelated anti-human CABYR 3A4 mAb used as a negative control antibody for flow cytometry and cytotoxicity assays, was developed in mouse using an immunogen as described earlier [27]. Non-specific mouse IgGs were used as a negative antibody control for IHC and IIF (Cell Signaling Technology, Danvers, MA). EpCAM (CD326) antibody (Miltenyi Biotec, San Diego, CA) was used as a positive antibody control for the cytotoxicity assays.

Fab’-specific peroxidase labeled secondary antibodies (Jackson ImmunoResearch, West Grove, PA), raised in goat to recognize mouse, were used for IHC (GtαMs HRP). Goat anti-mouse Alexa Fluor 488 (GtαMs Alexa488; Molecular Probes, Eugene, OR) was used to label primary antibodies in IIF. Goat anti-mouse R-phycoerythrin (GtαMs R-PE; Molecular Probes, Eugene, OR) was used as a secondary antibody for the flow cytometry assay. Fab-anti-mouse IgG Fc region-duocarmycin DM antibody with a cleavable linker (Fab-CL-DMDM; Moradec, San Diego, CA) was used as the secondary antibody-drug conjugate in the cytotoxicity assays.

Nickel-NTA agarose purified recombinant human SAS1B protein as described by Pires *et al*. [14] was used to immunoabsorb both 6B1 and SB2 mAbs in the IHC and flow cytometry assays. A SAS1B N-terminal peptide was used to immunoabsorb SB2 mAb in the cytotoxicity assay and a SAS1B peptide matching the C-terminus, not recognized by SB2, was used as a negative control peptide. SAS1B peptides were purified greater than 95% by analytic HPLC (Atlantic Peptides, Lewisburg, PA). SAS1B N-terminal peptide sequence was APLASSCAGACGTSFPDGL and the C-terminal peptide sequence was GAPGVAQEQSWLAGV.

### Cell lines and culture conditions

mPanc96 cells were obtained from the American Type Culture Collection (ATCC; Manassas, VA) while 608 and 366 fresh patient-derived cell lines were obtained as described previously [28, 29]. Primary keratinocytes, isolated from neonatal foreskin following a previously described protocol, were kindly provided by Dr. S.B. Vande Pol (University of Virginia, Department of Pathology,) [30, 31]. Pancreatic cancer cells were cultured in RPMI (Invitrogen, Carlsbad, CA) containing 10% heat inactivated fetal bovine serum (FBS; ThermoFisher Scientific, Waltham, MA) and 1% penicillin-streptomycin (Invitrogen, Carlsbad, CA). Keratinocytes were cultured in keratinocyte serum free medium (KSFM; Invitrogen, Carlsbad, CA) containing 1% penicillin-streptomycin. Cells were cultured in a humidified incubator (37°C, 5% CO_2_). mPanc96 cells were authenticated before purchase by the ATCC with cytochrome *c* oxidase subunit 1 analysis, DNA profiling, cytogenetic analysis, flow cytometry, and immunocytochemistry. Cell lines 608 and 366 were authenticated in 2010 and 2011 by the University of Virginia Biomolecular Research Facility with DNA profiling, cytogenetic analysis, flow cytometry, and immunocytochemistry.

### Human tissue microarray and IHC

The pancreatic progression TMA was provided by the Cooperative Human Tissue Network, funded by the National Cancer Institute (NCI) (available at: http://chtn.sites.virginia.edu/tissue-microarrays). Other investigators may have received specimens from the same subjects.

IHC was performed using the protocol as described previously [16], with some modifications. Histology sections were deparaffinized by melting and clearing in xylene followed by rehydration in descending grades of alcohol. Antigen retrieval with citrate buffer was performed by microwaving for 20 minutes followed by blocking with 5% non-fat dry milk (NFDM) containing 5% normal goat serum (NGS; Sigma, St. Louis, MO) in PBS for one hour at room temperature. Anti-SAS1B mAb, 6B1, or mouse IgG’s were applied to slides overnight at 4°C at 10 μg/mL. For the immunoabsorption assay, 6B1 mAb was pre-incubated with forty-times excess of rSAS1B protein for one hour prior to addition to the tissue and then sections were otherwise treated in the same manner. Slides were washed, quenched in methanol-hydrogen peroxide, incubated with 1:500 dilution of GαM HRP for one hour at room temperature, and then washed. Development of brown reaction product was then performed using 3, 3-diaminobenzidine (Sigma, St. Louis, MO). Hematoxylin was used as a counterstain then slides were dehydrated in increasing grades of alcohol. Slides were air-dried, mounted, and then imaged with an Olympus BX51 (Center Valley, VA). A board-certified pathologist (AMM) reviewed and scored all slides.

### Tissue processing for RNA and Protein

Cell lysis was performed on flash frozen PDX PDAC tumor samples and normal pancreas using the SuperFastPrep-1 with lysing Matrix D tubes (MP Biomedicals, Santa Ana, CA) in a 4°C cold room. RNA was then purified with the RNeasy kit (Qiagen, Germantown, MD) and reverse transcribed to cDNA as described previously [16]. PDX PDAC samples used for IHC were fixed and embedded as detailed earlier [22].

### Primers and RT-PCR

Primers designed to amplify the C-terminus of SAS1B, or GAPDH as a control, were used in a PCR assay, both previously described [16]. PCR products were run on a 1% agarose gel containing ethidium bromide and bands of the correct size were excised. cDNA was gel purified (QIAquick PCR Purification Kit, Qiagen, Germantown, MD) and sub-cloned using TOPO-TA cloning kit (Invitrogen, Carlsbad, CA). Plasmid DNA was purified (QIAprep Spin Miniprep Kid, Qiagen, Germantown, MD). DNA was sequenced and then searched with BLAST (NCBI) to confirm *ASTL*/SAS1B identity.

### Indirect immunofluorescence (IIF)

#### Fixed and permeabilized IIF

Cells were grown for 30–40 hours on coverslips which were first pre-incubated with fibronectin (Sigma, St. Louis, MO) at a 1:2000 dilution. Cells were then fixed with 4% paraformaldehyde (PFA) in DPBS for 15 minutes at room temperature. After washes with DPBS, cells were blocked and permeabilized with 5% heat-inactivated NGS in DPBS containing 0.1% Triton X100 for 30 minutes at 37°C. Anti-SAS1B mAb, SB2, or mouse IgGs were added to coverslips at a concentration of 6 μg/mL and incubated at room temperature for 1.5 hours. Following washes, a 1:500 dilution of GtαMs Alexa488 secondary antibody plus a 1:1000 dilution of DAPI was added to coverslips for one hour in the dark. Coverslips were washed, mounted with ProLong Gold Antifade (Invitrogen, Carlsbad, CA), dried, and imaged using a LSM 700 laser scanning confocal microscope (Zeiss, Oberkochen, Germany).

### Live and non-permeabilized IIF

Cells were grown on fibronectin coated coverslips for 30–40 hours and then blocked with 5% heat-inactivated NGS in media for 30 minutes at room temperature. Coverslips were then incubated with media containing 0.1% sodium azide (NaN_3_) for 30 minutes on ice. SAS1B mAb, SB2, or mouse IgGs were added to coverslips at a concentration of 10 μg/mL and incubated on ice for 1.5 hours and then washed. A 1:500 dilution of GtαMs Alexa488 secondary antibody was added to coverslips for one hour in the dark. Coverslips were washed, fixed with 4% PFA-DPBS at room temperature for 15 minutes, washed again, then stained with a 1:1000 dilution of DAPI. Following additional washes, coverslips were processed as described in fixed IIF.

### Low cytometry

Cells were grown to 80% confluency then dissociated with StemPro Accutase (Invitrogen, Carlsbad, CA). Cells were resuspended in media and allowed to recover at 37°C, 5% CO_2_ for two hours, with intermittent shaking of the tubes. Cells were blocked with media containing 0.1% NaN_3_ and 5% heat-inactivated NGS (referred to as “blocking media”) for 30 minutes on ice. SAS1B mAb, SB2, or unrelated, negative control mAb, 3A4, were added to cells at a concentration of 10 μg/mL made in blocking media and incubated on ice for two hours. For the immunoabsorbed sample, SB2 was pre-incubated with forty-times excess rSAS1B protein at room temperature for one hour prior to addition to cells. EpCAM was used as a positive control antibody at a concentration of 1.0 μg/mL. Following washes, a 1:500 dilution of GtαMs R-PE made in blocking media was added to cells and incubated on ice, in the dark, for one hour. After washing, cells were resuspended in DPBS containing 1:1000 DAPI to distinguish live from dead cells. Acquisition and analysis were performed in the UVA Flow Cytometry Core Facility using a FACS Calibur flow cytometer (BD Biosciences, San Jose, CA) and FlowJo software, version 9.8.2 (FlowJo, Ashland, OR).

### Cell line cytotoxicity

Cells were grown to 80% confluency, dissociated with Accutase, then plated in a 96 well plate and incubated overnight. Fab-CL-DMDM was pre-incubated with primary antibodies SB2, 3A4, or EpCAM made in cell media, in 1:10 serial dilutions, to allow primary antibodies to complex with secondary-drug conjugates for one hour at room temperature. For the immunoabsorption assay, SB2 mAb was pre-incubated with five-times excess SAS1B peptide, either blocking peptide or negative control peptide made of an irrelevant SAS1B sequence, overnight at 4°C before mixing with secondary-drug conjugate. Final concentration of Fab-CL-DMDM added to cells was 15 nM and concentrations of primary antibodies ranged from 0.0016 μg/mL (0.01 nM) to 16 μg/mL (100 nM). Cells were incubated with ADCs for 72 hours. Following incubation, media was removed from each well and 1X CellTiter-Glo 2.0 (Promega, Madison, WI) reagent in PBS was added and then incubated at room temperature for 10 minutes. ATP level of live cells was measured at room temperature using the BioTek Cytation3 luminometer. Percent cell viability was calculated by dividing luminescence values of ADC treated cells by the baseline luminescence value obtained from averaging cells which received only Fab-CL-DMDM. Triplicate experiments were performed independently, each with 3 technical repeats. The LD50s were calculated for each cell line and analyzed for statistical significant using non-linear regression (*p*-value < 0.05). An ANOVA, Student *t*-test was used to determine if the difference in cytotoxicity between pancreatic cancer cell lines and keratinocytes was statistically significant (*p*-value < 0.05).

## SUPPLEMENTARY MATERIALS FIGURES



## Abbreviations

ADC: antibody-drug conjugate
ASTL: astacin-like metallo-endopeptidase
ATCC: American Type Culture Collection
CABYR: calcium binding tyrosine phosphorylation regulated
CAR-T: chimeric antigen receptor T cells
CGA: cancer germline antigen
CTA: cancer testis antigen
DAPI: 4’,6-diamidino-2-phenylindole
DPBS: Dulbecco’s phosphate-buffered saline
EpCAM: epithelial cell adhesion molecule
Fab-CL-DMDM: Fab (fragment antigen binding) anti-mouse IgG Fc (fragment constant) region duocarmycin DM
FDA: food and drug administration (United States)
GtαMs Alexa488: goat anti-mouse Alexa Fluor 488
GtαMs HRP: goat anti-mouse horseradish peroxidase
GtαMs R-PE: goat anti-mouse R-phycoerythrin
HPLC: high-performance liquid chromatography
IgG: immunoglobulin G
IHC: immunohistochemistry
IIF: indirect immunofluorescence
LD50: lethal dose 50%
mAb: monoclonal antibody
NFDM: non-fat dry milk
NGS: normal goat serum
Nickle-NTA: nickel-nitrilotriacetic acid
PanIN: pancreatic intraepithelial neoplasia
PDAC: pancreatic ductal adenocarcinoma
PDX: patient derived xenograft
PFA: paraformaldehyde
RPMI: Roswell Park Memorial Institute medium
rSAS1B: recombinant SAS1B protein
RT-PCR: reverse transcription polymerase chain reaction
SAS1B: sperm acrosomal SLLP1 binding
SAS1B^neg^: SAS1B negative
SAS1B^pos^: SAS1B positive
SLLP1: sperm lysozyme-like protein 1
TMA: tissue microarray
UVA: University of Virginia
ZP2: zona pellucida protein 2
