# Supplementary material for: Evaluation of SAS1B as a target for antibody-drug conjugate therapy in the treatment of pancreatic cancer
Source: Oncotarget. 2018 Jan 4;9(10):8972–84. doi: 10.18632/oncotarget.23944 (PMC5823626; doi:10.18632/oncotarget.23944)
Supplement: Supplementary file 1 [file oncotarget-09-8972-s001.pdf]

Evaluation of SAS1B as a target for antibody-drug conjugate therapy in the treatment of pancreatic cancer

SUPPLEMENTARY MATERIALS

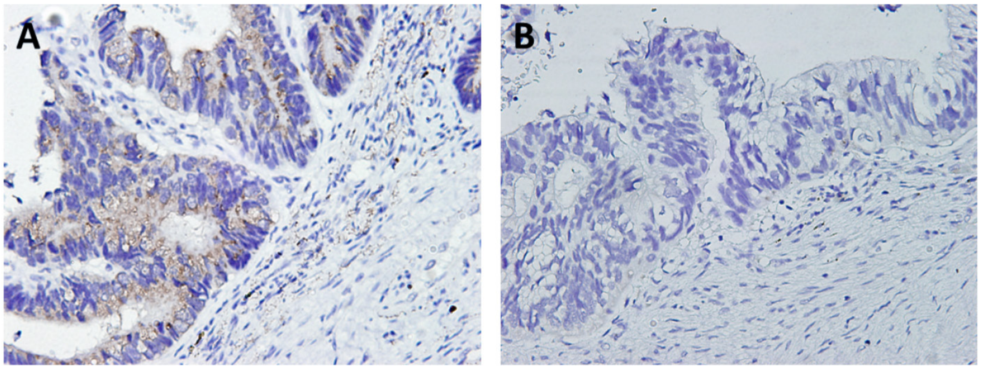

Supplementary Figure 1: SAS1B signal in pancreatic cancer tissue was blocked by pre-incubating anti-SAS1B mAb, 6B1, with rSAS1B protein. (**A**) Human primary PDAC tumor stained with anti-SAS1B mAb, 6B1, showed robust cytoplasmic signal in the tumor and trace staining in adjacent stromal cells. (**B**) SAS1B signal in tumor and stroma was blocked when anti-SAS1B mAb 6B1 was pre-incubated with 40× excess rSAS1B protein for one hour before addition to the tissue section. (B) is a serial section of (A). Images are 400x magnification.

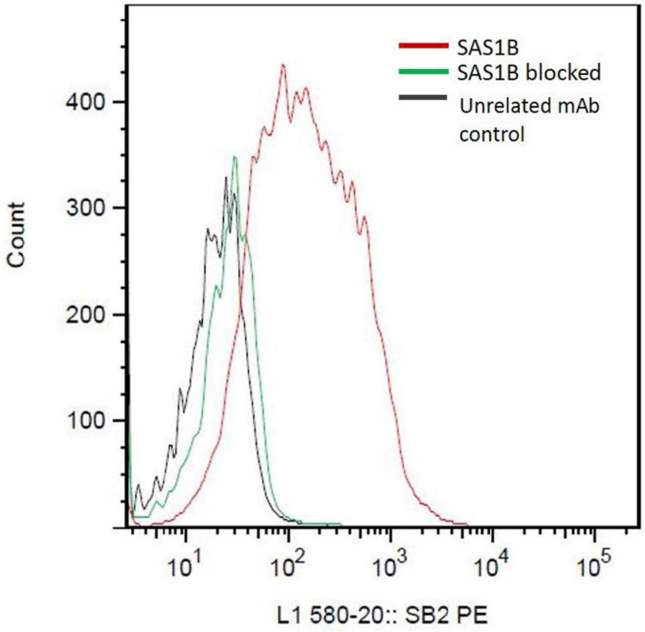

Supplementary Figure 2: Anti-SAS1B mAb, SB2, signal in live mPanc96 cells was blocked when pre-incubated with rSAS1B protein. Cell surface SAS1B detected by live cell flow cytometry in mPanc96 cells using anti-SAS1B mAb, SB2 (red line) as compared to unrelated mAb control (black line). When SB2 was pre-incubated with 40× excess rSAS1B protein, then added to cells, SAS1B signal was effectively blocked (green line).

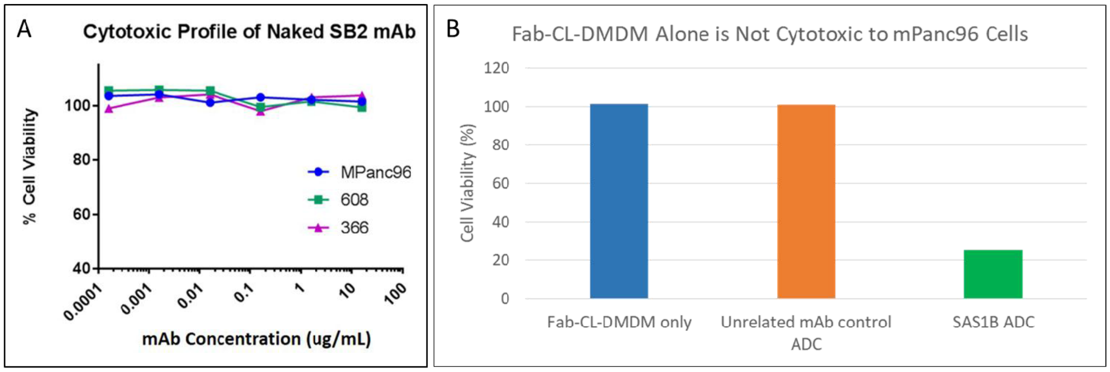

Supplementary Figure 3: Neither SB2 alone nor Fab-CL-DMDM were cytotoxic to pancreatic cancer cell lines. (**A**) No cytotoxic effects were observed in pancreatic cancer cell lines mPanc96, 608, and 366 when anti-SAS1B mAb, SB2, alone (no drug-conjugate) was added to cells. (**B**) Secondary-drug conjugate (Fab-CL-DMDM) showed no cytotoxic effects on cells. Representative experiment shown in mPanc96 cells. For both assays, SAS1B mAb incubated with cells for 72 hours. Relative cell viability measured using CellTiter-Glo.

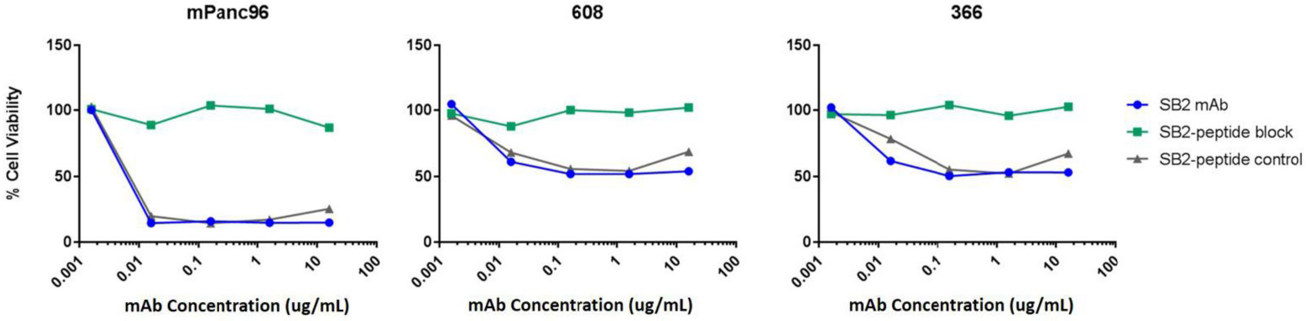

Supplementary Figure 4: Cytotoxicity of an anti-SAS1B ADC was blocked in pancreatic cancer cell lines when SB2 mAb was pre-incubated with a SAS1B peptide. Cytotoxicity induced by anti-SAS1B ADC (SB2 mAb) (blue line) is blocked when SB2 mAb is immunoabsorbed with SAS1B peptide (green line) in pancreatic cancer cell lines mPanc96 [left], 608 [middle] and 366 [right]. Cytotoxicity is not blocked when SB2 is immunoabsorbed with a negative control peptide (grey line) consisting of an irrelevant SAS1B sequence. SAS1B ADC incubated with cells for 72 hours. Relative cell viability measured using CellTiter-Glo.

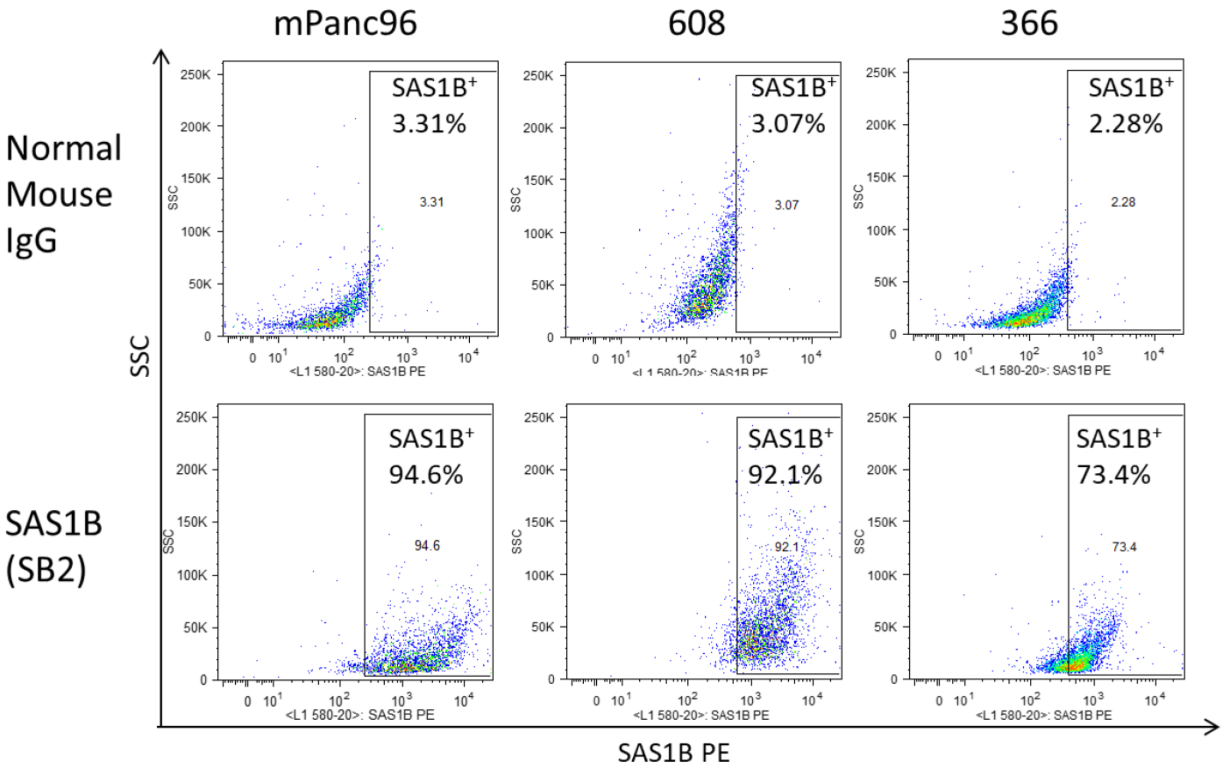

Supplementary Figure 5: SAS1B was expressed in a majority of fixed and permeabilized PDAC cells by flow cytometry. Intracellular SAS1B detected in fixed and permeabilized mPanc96 (left), 608 (middle), and 366 (right) cell lines by flow cytometry with SB2 monoclonal antibody (bottom row) or negative control normal mouse IgGs (top row). Data are representative of three experiments.
